# Assessment of Diameter Stability in Morse Taper Dental Implants with Different Angulations After Abutment Connection

**DOI:** 10.3390/ma18143403

**Published:** 2025-07-21

**Authors:** Bruno Q. S. Cordeiro, Waldimir R. Carvalho, Edgard M. Fonseca, Aldir N. Machado, Bruna Ghiraldini, Michel A. D. Soares, Priscila L. Casado

**Affiliations:** 1 Post-Graduation in Dentistry Program, School of Dentistry, Fluminense Federal University (UFF), Niterói 24020140, RJ, Brazil; brunoqsc@hotmail.com; 2 Prostheses Department, School of Dentistry, Fluminense Federal University (UFF), Niterói 24020140, RJ, Brazil; wcarvalho@id.uff.br (W.R.C.); edgardfonseca@id.uff.br (E.M.F.); 3 Clinical Center of Research in Implant Dentistry, School of Dentistry, Fluminense Federal University (UFF), Niterói 24020140, RJ, Brazil; aldirmachado@id.uff.br; 4 Paulista University (UNIP), São Paulo 03166001, SP, Brazil; bruna.ghiraldini@sinimplantsystem.com (B.G.); michel.soares@sinimplantsystem.com.br (M.A.D.S.); 5 Post-Graduation in Implant Dentistry Program, School of Dentistry, Fluminense Federal University (UFF), Niterói 24020140, RJ, Brazil

**Keywords:** dental implants, Morse connection, prosthetic torque, plastic deformation

## Abstract

Background: Modification of diameter stability after the abutment retention can result in a decrease in the applied torque or affect the peri-implant tissue, compromising the longevity of the treatment. Therefore, this study aimed to investigate how different connection angles (11.5° and 16.0°) at the implant–abutment interface influence implant diameter stability under the manufacturer’s recommended torque. Methods: Eighty Morse cone-type implant specimens were divided into two groups, with different internal conicity angles: 11.5° (*n* = 40) and 16.0° (*n* = 40). Implants varied in diameter (mm): 3.5, 3.8, 4.5, and 5.0. Initial measurements of the implants’ external diameter were carried out. After these measurements, all implants received the abutment installation, and a final measurement of the external implant diameter was performed. Results: Considering the comparative analysis between the final and initial diameters, a non-significant increase in diameter, in the cervical implant region, after torque on the abutment, was observed. The torque applied to the abutments did not produce deformations in the cervical area of Morse taper implants. Conclusions: The torque applied to the abutment screw in implants with a Morse taper connection does not cause deformation in the cervical area of the implant body in implant with 11.5° and 16.0° conicity angles.

## 1. Introduction

Contrary to natural teeth, dental implants are directly in contact with the bone. The bone–implant interface exhibits the lowest resistance level, as occlusal loads are transmitted directly to the bone’s periphery. This contrast becomes evident when examining the dynamics of occlusal load on the implant because natural teeth have a periodontal ligament, which allows stress absorption and movement within the alveolus [1]. Various factors such as the type of loading, supporting bone tissue, implant geometry, and the mechanical properties of both the implant and the prosthesis influence this behavior. If the occlusal force surpasses the absorption capacity of the osseointegrated interface, the implant is prone to failure [2].

Dental implant systems vary in the macrogeometry of the implant and the geometry of the implant–abutment interface. These include external connections (with an external hexagon pattern on the implant platform), internal connections (which have various morphologies such as internal hexagon, internal octagon, and internal trigon), and internal conical connections with different angles at the implant–abutment interface [3,4].

In 1864, the Morse cone connection was developed by Stephen A. Morse [5,6], and the basic principle of this system was “a cone within a cone,” simulating the implant–abutment interface [7]. In dentistry, in addition to the mechanical locking of the Morse connection, screw retention was added to this system [8].

In vitro studies have demonstrated that internal connections are more mechanically stable and appear to play an important role in the loss of preload and screw loosening compared to flat external connections (external hexagon type), indicating a superiority of internal connections [9,10]. However, abutment screws must be tightened during insertion according to the torque recommended by the manufacturer, and for this, torque wrenches are essential devices to ensure adequate torque and prevent screw loosening [11,12].

Complications in osseointegrated implants can manifest themselves biologically and mechanically, with the loosening of the abutment retention screw. This phenomenon can be a result of a decrease in the applied torque, which can be caused by a series of factors or their interaction [13], including torque variation produced by different mechanical devices, geometric design of the implant, type of alloy and screw surface, in addition to the constant compressive load on the pillar screw, and variables induced by the operator [11].

A recent study associated screw loosening with the type and height of the implant abutment, associating a stock abutment with a post height of 4 mm with effectiveness in minimizing screw loosening [14]. In addition, a previous study showed that the manual torque applied in laboratories may differ from the torque suggested by the manufacturer, which may alter the total height of prosthetic abutments in Morse taper implants. These numerous variations in abutment heights, study design, and clinical or laboratory torque can affect the outcome of long-term success [15].

Another important consideration highlighted by Gehrke et al. [16] is that high installation torque values can cause irreversible mechanical damage to implants. However, no study to date has clearly associated potential damage to implants with precise changes in shape, strength, survival, or load distribution [14]. However, it is a fact that loosening of abutment or prosthesis screws can induce problems, such as disturbances in the transfer and distribution of applied occlusal forces, screw or implant fracture, and microgap formation at the implant–abutment interface, allowing bacterial infiltration and affecting osseointegration [17].

This interface is the weak link in the dental implant system, needing to withstand maximum and continuous masticatory forces, as well as prevent bacterial penetration, which could otherwise lead to increased marginal bone loss [4]. The interaction between abutment insertion and implant diameter deformation is a crucial aspect of dental implant mechanics. When an abutment is inserted into an implant, the connection must be secure to ensure the longevity and stability of the implant. However, this process can lead to mechanical complications:

- deformation at the implant–abutment interface: repeated tightening and retightening of the abutment can cause deformation at the interface, potentially leading to microleakage and loss of preload;
- plastic deformation: high-frequency loading and cyclic forces can induce plastic deformation of the implant shoulder, which compromises the integrity of the implant;
- mechanical failures: such issues as screw loosening, abutment or implant fractures, and wear at the implant–abutment interface are common mechanical failures associated with these deformations [8].

While previous research highlighted the mechanical stability of internal connections, the impact of different implant–abutment connection angles on diameter stability remains underexplored. Variations in connection angles could influence the mechanical resistance and overall performance of dental implants, potentially affecting clinical outcomes. In addition, according to the literature, different angles at the implant–abutment interface may result in differences in mechanical resistance and biomechanical behavior because of the varying contact surfaces between the implant and the prosthetic abutment. The implant–abutment connection is vital for the long-term success of dental implant rehabilitation, as it is significantly involved in all biological and technical complications [14,18,19].

Despite these advances, the literature has not yet comprehensively investigated the plastic deformations in the implant body caused by the insertion torque of the abutment. Therefore, the aim of this study was to investigate how different connection angles at the implant–abutment interface influence implant diameter stability under the manufacturer’s recommended torque. Understanding these deformations is critical for optimizing implant design and ensuring the long-term success and reliability of dental implants. The initial hypothesis was that the different internal angles, 11.5° and 16.0°, do not influence the alteration of the implant diameter. Clinically, elucidating these effects could lead to improved implant systems that better withstand occlusal forces, thereby reducing complications such as screw loosening and bacterial infiltration. Scientifically, this study contributes to the broader field of implant biomechanics by addressing a gap in the literature, offering insights that could refine current practices and protocols in implant dentistry.

## 2. Materials and Methods

This research is of a technical laboratory nature, not involving the participation of humans or animals or any procedure that includes biological material or personal data; therefore, it was not necessary to submit this research for evaluation to a research ethics committee.

**Figure 1:**
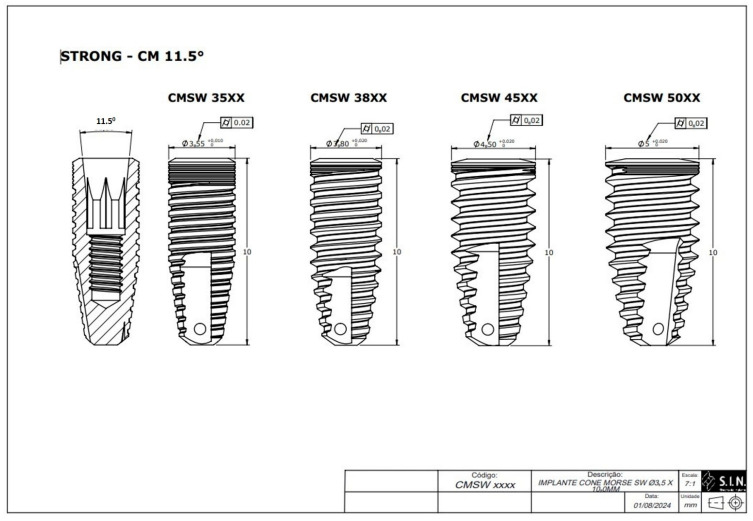
Technical measurements of the Morse taper implants with an 11.5° internal taper (S.I.N. Implant System, São Paulo, SP, Brazil).

**Figure 2:**
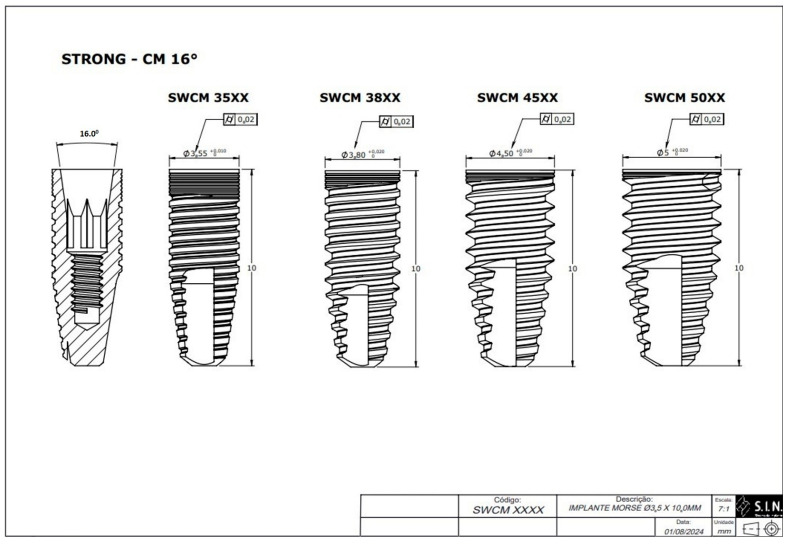
Technical measurements of the Morse taper implants with a 16° internal taper (S.I.N. Implant System, São Paulo, SP, Brazil).

Research instrument: Eighty specimens were manufactured (*n* = 80) using 40 implants with an internal conicity of 11.5° and 40 implants with an internal conicity of 16.0° (Strong SW, S.I.N. Implant System, São Paulo, SP, Brazil). All the implants had the same length (10 mm) but varied in diameter. The specimens were divided into eight groups, with 10 specimens of each diameter in each group for repeated analysis (Table 1, Figure 1 and Figure 2). Diameter differences of up to 0.02 mm were considered within the normal deviation standard during the implant manufacturing process, based on the manufacturer’s technical assessment and quality standards.

**Figure 3:**
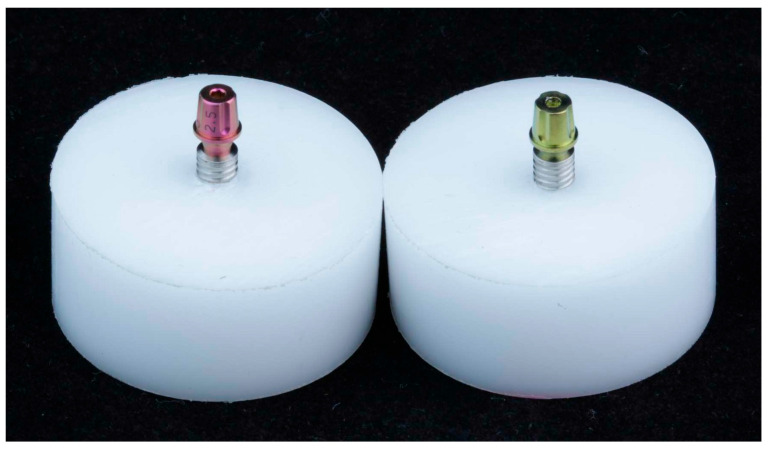
Test specimens used in the study. (**Left**) Connection from Morse cone implants with an 11.5° internal conicity. (**Right**) Connection from Morse cone implants with a 16° internal conicity.

All the implants were placed in polyacetal resin cylinders, with a density of 1.42 g/cm^3^, with an elastic modulus greater than 3 GPa (polyacetal, Caterplast, São Paulo, Brazil), with a torque of 40 N·cm (insertion torque recommended by the manufacturer was 30–60 N·cm, S.I.N. Implant System, São Paulo, Brazil), leaving 3 mm of exposure for these implants, as recommended by ISO 14801 [20] (Figure 3). ISO 14801 was chosen for this study as it is the standard that specifies the dynamic fatigue testing methodology for endo-osseous dental implants, which ensures consistency and reliability in mechanical testing. This standardization is crucial in replicating the clinical conditions under which implants might experience mechanical stress, ensuring the results are applicable to real-world scenarios. Polyacetal resin, with its known machinability and elastic modulus, provides a consistent and reliable medium to evaluate the mechanical behavior of implants under simulated clinical conditions. This choice ensures that the testing adheres to internationally recognized standards, thereby increasing the reliability and applicability of the findings.

**Figure 4:**
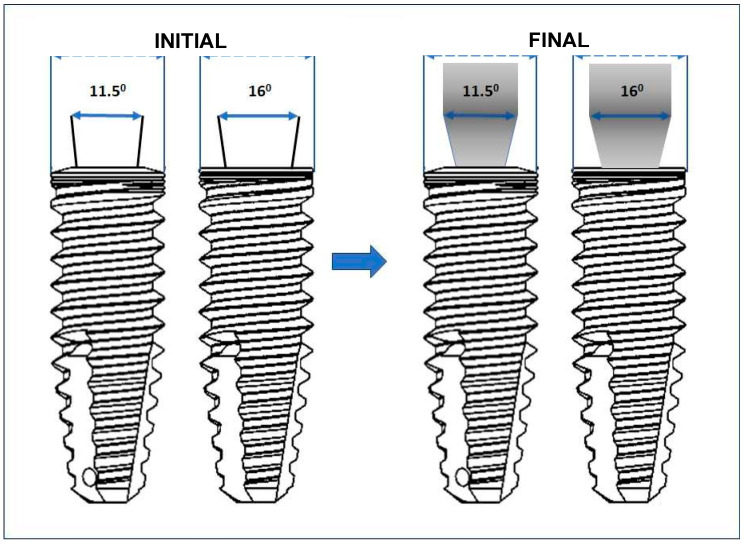
Schematic representation of the measurements of the initial (before connecting the prosthetic abutment) and final (after connecting the prosthetic abutment) diameters.

After preparing the test specimens, initial measurements of the external diameter of the implants were carried out. After these measurements, all the implants received the abutment (screwless) installation. The implants with 16.0° (*n* = 40) received cemented universal abutments (AIM 45402C, S.I.N. Implant System, São Paulo, Brazil), and the 11.5° implants (*n* = 40) received straight universal abutments (AISIT 454025, S.I.N. Implant System, São Paulo, Brazil), all with a torque of 20 N·cm (torque recommended by the manufacturer), using a universal torque wrench, and final measurements of the external diameter of the implant platform were performed for each group. These measurements were, therefore, carried out at 2 different times: initial—before installing the abutment; final—immediately after installing the pillar (Figure 4).

The specimens were subjected to a three-dimensional coordinate measuring machine, serial No. 70136290, certification No. 00361/23—RBC—Mitutoyo (Jundiaí, São Paulo, Brazil), measurement procedure PML-0048—Review: 4, measurement certificate No. 01977/23 from the Brazilian calibration network at a calibration laboratory accredited by Cgcre in accordance with ABNT NBR ISO/IEC 17025 under number CAL 0031 [21]. Multiple measurements were taken for each implant to ensure accuracy and reliability. These repeated measurements allowed for an average to be calculated, minimizing variability and enhancing the precision of the data collected. This approach addresses measurement variability by ensuring that any anomalies are balanced out across multiple readings. Data processing and statistical analysis were conducted using JASP 0.18.3 (Amsterdam University, Amsterdam, The Netherlands) and PRISMAGRAPH version 10.4.4. The Shapiro-Wilk statistical test to identify normal or non-normal distribution was applied to each group. The distribution in all the groups was non-normal, with *p* < 0.001. The Mann–Whitney U test was then used to verify possible differences between implant diameters.

## 3. Results

Analysis of the results comparing the measurements, in millimeters (mm), within each group showed that at an angulation of 11.5°, all the implants with 3.5, 3.8, 4.5, and 5.0 mm showed no significant differences in diameter after connecting the abutment, ranging from 0.001 to 0.003 mm (Table 2).

Taking into consideration the implants with 16°, the analysis of the results comparing the measurements, in millimeters (mm), within each group showed that the implants with 3.5, 3.8, 4.5, and 5.0 mm in diameter showed a non-significant increase in diameter after connecting the abutment, ranging from 0.001 to 0.004 mm (Table 3).

The results supported the initial hypothesis that the different internal angles, 11.5° and 16.0°, do not influence the alteration of the implant diameter.

Regarding the initial implant diameter, homogeneity in the manufacturing of the provided diameters was observed, aligning with the manufacturer’s reported diameter, considering differences of up to 0.02 mm as acceptable within the manufacturing threshold provided by the company.

## 4. Discussion

Internal conical connections have demonstrated better biomechanical behavior when compared to hexagonal connections [9,10]. However, internal conical connections have different configurations regarding the conicity of the connection angle. This study showed that 11.5° and 16.0° angulation of conicity of internal conical connections had no influence over modifications at the diameter size of the implant after abutment connection. In the present study, one internal conical implant prosthetic connection was used, but with different angles of conicity at the implant–abutment interface: 11.5° and 16.0°. The change in angulation of the implant–abutment interface does not lead to differences in the mechanical resistance and biomechanical behavior due to the difference in the contact surface between the interface of the implant and the prosthetic abutment. This finding suggests that clinicians can confidently use either angle without concern for increased risk of mechanical failure or fatigue, ensuring reliable long-term outcomes for prosthetic rehabilitations. The study reinforces that the choice between these angles can be based on other factors, as neither poses a disadvantage in terms of altering implant diameter or stability.

Morse taper dental implants offer several significant advantages, including the ability to minimize the microgap between the implant and the abutment. The conical shape of the connection favors the distribution of occlusal forces along the length of the implant, reducing stress concentration at the crestal bone level. This factor contributes to better osseointegration and bone preservation [22]. However, recent studies indicate that axial loading on Morse taper implants can result in deformations of both the internal and external walls of the implant, particularly in the cervical region, and this deformation is influenced by the implant diameter [23,24]. In our study, it was observed that the applied torque did not influence the deformation of the implant’s external wall regardless of diameter.

The accumulated preload on the abutment screw, which is crucial for the stability of the implant system, must be within a specific range relative to the material’s yield strength [25,26]. Correct torque application is essential to ensure the long-term stability of the implant–abutment interface, preventing future issues related to connection loosening. Such loosening can compromise the efficiency of load distribution along the axial axis of the implant and result in peri-implant bone loss.

The preload exerted on the abutment screw depends not only on the applied torque but also on the friction at contact surface interfaces [27]. Nissan et al. [18] reported that forces applied to the implant superstructure result in tensile and compressive stresses at the implant–abutment connections, leading to micromovements that can cause loss of screw torque. This can compromise the connection stability and increase the risk of failure [18,19]. In our study, the increase in the implant’s dimensions after torque application was associated with accurate positioning of the prosthetic connection, resulting in full contact between the prosthetic component and the internal face of the implant. This complete contact helps avoid gaps and facilitates efficient load transfer from the extraosseous to the intraosseous environment, preventing the formation of future gaps.

Elastic deformation of both the implant and the abutment can create a high-tension force between them. Using a Morse taper implant–abutment connection without screws can be advantageous, eliminating mechanical complications associated with retention screws and facilitating the rehabilitation of misaligned implants [28]. Screwless connections have been shown to withstand average occlusal forces even after prolonged periods of artificial loading, suggesting that these connections can offer stability and reduce maintenance needs.

Despite the advantages of screwless connections, it is important to recognize that a microgap can still form, even with a well-fitted connection [29]. Schmitt et al. [30] observed that no implant connection offers a complete seal at the implant–abutment interface. Comparisons show that microleakage in Morse taper connections is lower compared to external and internal hexagon connections [31].

The literature also suggests that a low friction coefficient on contact surfaces leads to a higher preload on the abutment screw compared to a high friction coefficient when the same torque is applied [32]. This was demonstrated by Guzaitis et al. [33], who showed that the reduction in friction coefficient was caused by changes in surface morphology due to repeated tightening and loosening of the screw.

Some authors have shown that higher torques reduce the linear contact area between the implant and the abutment, decreasing the gap between the parts. Gehrke et al. [16] indicated that a torque of 35 N·cm was the most suitable for internal conical connections (cone with 11°), but they did not assess changes in implant diameter. In our study, the application of a torque of 40 N·cm did not cause deformation in the diameter of the implant’s external wall, suggesting that the Morse connection can preserve the peri-implant bone profile and ensure the stability of the implant structure. This contributes to the preservation of peri-implant mucosal health, preventing gaps between the abutment and the implant that could be filled with bacterial biofilm.

The application of cyclic loading on Morse taper implants can lead to the accommodation of the implant–abutment connection, reducing or eliminating the gap between them [16]. This accommodation can increase friction between the walls without affecting the implant’s external wall diameter. We assume that increasing the implant’s diameter after torque application offers advantages by increasing the bone–implant contact without affecting the prosthetic and implant structure, playing a key role in the internal connection stability and the external peri-implant tissue stability. In our study, even when using wide implants with a 5.0 mm diameter, there was no damage to the implant’s mechanical and structural stability. Moreover, the statistical insignificance of these differences further ensures the stability of prosthetic rehabilitations when using abutments in 11.5° and 16.0° Morse taper connections, confirming that there is no modification in the implant diameter that could potentially influence failures, fatigue, or mechanical misalignment in the future.

Furthermore, clinical situations such as cantilevers, implant position and angulation in the arch, occlusal forces, and parafunctions can contribute to deformations in the implant body or loosening of abutment retention screws [34]. These variables can lead to non-axial distribution of forces and, consequently, peri-implant failures. Therefore, it is crucial to ensure the use of implants with larger diameters without the risk of structural modifications, which directly affect the biomechanics of the abutment–implant system.

As there are no specific data in the literature regarding the metric difference of deformation in the implant body after torque application, a direct comparison with other studies is not possible. However, it is widely accepted that torque application can increase stress levels in the implant [35,36,37]. In our study, it was observed that increasing the implant diameter did not cause structural deformation regardless of angulation. However, one limitation of the study is that it was conducted in a laboratory setting, which cannot perfectly replicate the complex and variable conditions of the human oral cavity. It also does not account for biological variability among patients, such as bone density and masticatory load, which can influence implant behavior.

## 5. Conclusions

This study concluded that the torque applied to the abutment screw in implants with a Morse taper connection does not cause deformation in the cervical area of the implant body, in implants with 11.5° and 16.0° conicity angles. The change in angulation of the implant–abutment interface does not lead to differences in the mechanical resistance and biomechanical behavior. This finding suggests that clinicians can confidently use either angle without concern for increased risk of mechanical failure or fatigue, ensuring reliable long-term outcomes for prosthetic rehabilitations. For future research, exploring long-term clinical studies, diverse implant systems, and advanced imaging techniques could enhance understanding and refine clinical guidelines.

## Figures and Tables

**Table 1:** Groups of 10 mm long implants, divided according to the internal conic angle and implant diameter (mm). Note: * identification is based on the specific diameter for each internal taper.

Group (Internal Taper)	Implant Diameter (mm) (*n* = 10 Each)	Specimen ID *	Code
11.5°	3.5	1	CMSW3510
	3.8	2	CMSW3810
	4.5	3	CMSW4510
	5.0	4	CMSW5010
16°	3.5	5	SWCM3510
	3.8	6	SWCM3810
	4.5	7	SWCM4510
	5.0	8	SWCM5010

**Table 2:** Differences between the initial and final measurements in Groups 1, 2, 3, and 4, representing the 11.5° implants.

Implant	*Diameter (mm)*			
**CMSW 3.5 × 10** **11.5**°	**No torque**	**With torque**	**Difference**	***p*-value**
1	3.532	3.533	0.001	
2	3.534	3.537	0.003	
3	3.532	3.532	0	
4	3.538	3.54	0.002	
5	3.532	3.535	0.003	**0.82**
6	3.532	3.533	0.001	
7	3.545	3.548	0.003	
8	3.533	3.536	0.003	
9	3.53	3.532	0.002	
10	3.537	3.539	0.002	
**Mean ± SD**	**3.534 ± 0.004**	**3.536 ± 0.004**		
**CMSW 3.8 × 10** **11.5°**	**No torque**	**With torque**	**Difference**	***p*-value**
**1**	**3.817**	**3.82**	0.003	
**2**	**3.813**	**3.815**	0.002	
**3**	**3.809**	**3.812**	0.003	
**4**	**3.814**	**3.817**	0.003	
**5**	**3.807**	**3.809**	0.002	**0.72**
**6**	**3.815**	**3.818**	0.003	
**7**	**3.811**	**3.814**	0.003	
**8**	**3.809**	**3.811**	0.002	
**9**	**4.521**	**4.523**	0.003	
**10**	**4.522**	**4.524**	0.001	
**Mean ± SD**	**3.811 ± 0.003**	**3.814 ± 0.003**		
**CMSW 4.5 × 10** **11.5**°	**No torque**	**With torque**	**Difference**	***p*-value**
1	4.519	4.521	0.002	
2	4.522	4.524	0.002	
3	4.517	4.519	0.002	
4	4.52	4.521	0.001	
5	4.521	4.522	0.001	**0.47**
6	4.52	4.521	0.001	
7	4.518	4.519	0.001	
8	4.519	4.521	0.002	
9	4.521	4.523	0.002	
10	4.522	4.524	0.002	
**Mean ± SD**	**4.510 ± 0.001**	**4.520 ± 0.001**		
**CMSW 5.0 × 10** **11.5**°	**No torque**	**With torque**	**Difference**	***p*-value**
1	4.997	4.998	0.001	
2	4.999	5.00	0.001	
3	5.024	5.025	0.001	
4	5.002	5.002	0.000	
5	4.995	4.996	0.001	**0.92**
6	5.022	5.022	0.000	
7	4.996	4.997	0.001	
8	4.999	4.999	0.000	
9	4.998	4.999	0.001	
10	4.998	4.999	0.001	
**Mean ± SD**	**5.003 ± 0.011**	**5.003 ± 0.010**		

**Table 3:** Differences between the initial and final measurements in Groups 5, 6, 7, and 8, representing the 16° implants.

	*Diameter (mm)*			
**SWCM 3.5 × 10** **16**°	**No torque**	**With torque**	**Difference**	***p*-value**
1	3.547	3.551	0.004	
2	3.546	3.551	0.005	
3	3.549	3.553	0.004	
4	3.55	3.554	0.004	
5	3.55	3.555	0.005	**0.23**
6	3.55	3.554	0.004	
7	3.548	3.552	0.004	
8	3.547	3.552	0.005	
9	3.525	3.528	0.003	
10	3.549	3.554	0.005	
**Mean ± SD**	**3.546 ± 0.007**	**3.550 ± 0.007**		
**SWCM 3.8 × 10** **16°**	**No torque**	**With torque**	**Difference**	***p*-value**
**1**	**3.809**	**3.813**	0.004	
**2**	**3.812**	**3.816**	0.004	
**3**	**3.811**	**3.816**	0.005	
**4**	**3.81**	**3.814**	0.004	
**5**	**3.808**	**3.804**	−0.004	**0.30**
**6**	**3.805**	**3.809**	0.004	
**7**	**3.81**	**3.815**	0.005	
**8**	**3.81**	**3.814**	0.004	
**9**	**3.812**	**3.816**	0.004	
**10**	**3.808**	**3.812**	0.004	
**Mean ± SD**	**3.809 ± 0.002**	**3.812 ± 0.003**		
**SWCM 4.5 × 10** **16°**	**No torque**	**With torque**	**Difference**	***p*-value**
1	4.497	4.499	0.002	
2	4.501	4.504	0.003	
3	4.499	4.502	0.003	
4	4.5	4.503	0.003	
5	4.501	4.504	0.003	**0.25**
6	4.499	4.503	0.004	
7	4.499	4.503	0.004	
8	4.498	4.501	0.003	
9	4.501	4.505	0.004	
10	4.501	4.503	0.002	
**Mean ± SD**	**4.490 ± 0.001**	**4.500 ± 0.001**		
**SWCM 5.0 × 10** **16°**	**No torque**	**With torque**	**Difference**	***p*-value**
1	4.995	4.998	0.003	
2	4.999	5.003	0.004	
3	4.999	5.002	0.003	
4	4.997	5.00	0.003	
5	4.995	4.998	0.003	**0.36**
6	5.007	5.008	0.001	
7	5.009	5.012	0.003	
8	4.999	5.003	0.004	
9	4.994	4.997	0.003	
10	5.009	5.012	0.003	
**Mean ± SD**	**5.000 ± 0.005**	**5.003 ± 0.005**		

## Data Availability

The original contributions presented in this study are included in the article. Further inquiries can be directed to the corresponding author.
